# Dynamic prediction during perception of everyday events

**DOI:** 10.1186/s41235-018-0146-z

**Published:** 2018-12-29

**Authors:** Michelle L. Eisenberg, Jeffrey M. Zacks, Shaney Flores

**Affiliations:** 1 Washington University in St. Louis, Sierra Pacific MIRECC, VAPAHCS, Stanford University School of Medicine, 3801 Miranda Ave (151 Y), BLDG 5, 4th Floor, Palo Alto, CA 94304 USA; 2 Department of Psychological & Brain Sciences, Washington University in St. Louis, Campus Box 1125, 1 Brookings Drive, St. Louis, MO 63130 USA; 3 Department of Radiology, Washington University School of Medicine, Campus Box 8225, 4525 Scott Ave., St. Louis, MO 63110 USA

**Keywords:** Prediction, Event cognition, Eye tracking

## Abstract

The ability to predict what is going to happen in the near future is integral for daily functioning. Previous research suggests that predictability varies over time, with increases in prediction error at those moments that people perceive as boundaries between meaningful events. These moments also tend to be points of rapid change in the environment. Eye tracking provides a method for noninterruptive measurement of prediction as participants watch a movie of an actor performing a series of actions. In two studies, we used eye tracking to study the time course of prediction around event boundaries. In both studies, viewers looked at objects that were about to be touched by the actor shortly before the objects were contacted, demonstrating predictive looking. However, this behavior was modulated by event boundaries: looks to to-be-contacted objects near event boundaries were less likely to be early and more likely to be late compared to looks to objects contacted within events. This result is consistent with theories proposing that event segmentation results from transient increases in prediction error.

**Electronic supplementary material:**

The online version of this article (10.1186/s41235-018-0146-z) contains supplementary material, which is available to authorized users.

## Significance statement

The ability to predict what will happen in the near future is integral for adaptive functioning, and although there has been extensive research on predictive processing, the dynamics of prediction at the second by second level during the perception of naturalistic activity has never been explored. The current studies therefore describe results from a novel task, the Predictive Looking at Action Task (PLAT), that can be used to investigate the dynamics of predictive processing. Demonstrating the utility of this task to investigate predictive processing, it was applied to study the predictions made by Event Segmentation Theory, which suggests that people experience event boundaries at times of change and unpredictability in the environment. The results of these studies are of interest to communities investigating the dynamic comprehension and segmentation of naturalistic events and to communities studying visual perception of naturalistic activity.

## Introduction

The ability to anticipate what is going to happen in the near future is essential for survival. Prey animals must make predictions about the locations of their predators in order to avoid being eaten. Predators must anticipate the location of their prey so as not to starve. Predictive processing has been shown to play a central role in functions ranging from object recognition (e.g., Bar et al., 2006) to action guidance (e.g., Grush, 2004) to deliberative decision-making (e.g., Doya, 2008). Across these domains, humans and other organisms form representations that correspond to what is likely to happen in the near future.

One feature of everyday activity is that predictability varies over time such that at some times predictions are accurate and prediction error is low, whereas at other times prediction errors can spike suddenly. For example, when cooking a hotdog on a fire, it is easy to predict what is going to happen for the first few minutes: the hotdog will slowly become warmer and warmer as it begins to cook. However, at a certain point, the hotdog will quickly begin to turn brown, and it is difficult to predict exactly when it will go from browning to burning or even catching on fire.

Contemporary models have proposed accounts of how this variability in predictability is used by the perceptual system to enable adaptive processing of sequences of human activity. One recent theory, Event Segmentation Theory (EST; Zacks, Speer, Swallow, Braver, & Reynolds, 2007), describes the temporal dynamics of predictive processing in everyday event comprehension. EST proposes that people maintain working memory representations of the current event, *event models*, that inform perceptual predictions about future activity. These predictions are compared with sensory inputs from the environment to calculate a prediction error—the difference between the prediction and what actually occurs. When prediction error rises transiently, the working memory representation is updated to better reflect the current environment. In naturalistic activity, prediction error increases tend to happen when features in the environment are changing rapidly. For example, when watching someone prepare for a party, people would experience low levels of prediction error as the actor walks around the table and sets plates in front of each seat, because it is easy to predict that the actor is going to continue setting the table. However, when the actor finishes setting the table, there are many possible actions in which the actor could engage, and prediction error would increase as the actor switches to blowing up balloons. This increase in prediction error would cause updating of viewers’ event models to better represent the actor’s new goal of blowing up the balloons, causing people to experience a subjective event boundary. Throughout this process, predictions would continue to be made based on each new piece of information gathered from the environment. As the actor continues to blow up balloons, prediction error would decrease and the cycle would begin again.

EST relates the computational mechanism of prediction error-based updating to the subjective experience of events in a sequential stream of behavior. When an event model is updated, the perceiver experiences that one event has ended and another has begun. The subjective experience of event boundaries can be studied using a unitization task, in which participants are asked to press a button whenever they believe one meaningful activity has ended and another has begun (Newtson, 1973). Using boundaries defined with this technique, Zacks, Kurby, Eisenberg, and Haroutunian (2011) tested the hypothesis that event boundaries correspond to points of high prediction error. They asked participants to watch movies of an actor doing everyday activities (i.e., washing a car, putting up a tent). These movies were paused periodically and participants made predictions about what would occur five seconds later in the movies. The movies were then restarted and participants received feedback about the accuracy of their predictions by continuing to watch the movies. Some of the pauses occurred immediately before a boundary between activities such that the to-be-predicted activity was part of a new event, and some of the pauses occurred within activities such that the to-be-predicted activity was part of the current event. The authors found that predictions were less accurate when predictions were made across event boundaries than when predictions were made within events. Further, a functional MRI experiment described in the same paper demonstrated that structures in the midbrain associated with signaling prediction error were more activated when participants attempted to predict across an event boundary. These results provide evidence that prediction failures are associated with the perception of event boundaries. However, this study was limited, first, in that comprehension was stopped repeatedly to administer the prediction task and, second, in that it provided very little information about the temporal dynamics of prediction error.

Other relevant data come from studies using a narrative reading paradigm (e.g., Speer & Zacks, 2005; Speer, Zacks, & Reynolds, 2007; Zacks, Speer, & Reynolds, 2009b). In these studies, event boundaries tended to be identified at points when many features of the situation were changing, consistent with the suggestion that in naturalistic activity periods of change tend to produce prediction errors. Participants’ reading times slowed at event boundaries, and when readers were asked to rate the predictability of each clause they rated event boundaries as less predictable. Pettijohn and Radvansky (2016) showed that editing the text to make a feature change predictable eliminated slowing in reading time, consistent with the idea that event boundaries are associated with spikes in prediction error.

For the visual comprehension of naturalistic everyday activities, eye tracking provides a promising method for studying the time course of predictability. Eye tracking has been used to study predictive looking behavior in people ranging from infants to adults. For example, Haith and McCarty (1990) investigated infants’ anticipatory looks to stimuli that alternated between the left and right of the screen and found that infants made reliable anticipatory looks to the locations where stimuli were about to appear. In a similar study, Romberg and Saffran (2013) found that infants made anticipatory eye movements to locations where stimuli were probabilistically expected to appear. In addition, Hunnius and Bekkering (2010) studied predictive looking in infants using a paradigm in which the infants watched an actor use an object multiple times while the infants’ eyes were tracked using an eye tracker. On some trials, the actor used the object in a typical fashion (e.g., bringing a hairbrush to the head) and, on other trials, the actor used the object in an atypical fashion (e.g., bringing a hairbrush to the mouth). The authors found that infants predictively looked at the location where the object was typically used, even when the actor brought the object to the atypical location, meaning that the infants were not solely using motion information to make these predictions. Predictive looking has also been studied in adults. Flanagan and Johansson (2003), for example, had participants watch an actor move three blocks from one side of the table to the other while their eyes were tracked using an eye tracker. The authors found that participants started looking at the location where the blocks would be moved before the blocks arrived there, suggesting that participants were predicting the block movements. Similarly, Vig, Dorr, Martinez, and Barth (2011) found that when adults watched brief naturalistic scenes, eye movements to salient stimuli were nearly instantaneous, despite the fact that controlled laboratory studies have found that it takes an average of 200 ms to saccade to and fixate on a newly presented stimulus. The authors suggest that participants made predictive eye movements to the locations where salient information would soon be presented.

Predictive looking has also been studied extensively in the context of sports. For example, Hayhoe, McKinney, Chajka, and Pelz (2012) studied predictive eye movements as participants played squash. They found that participants made anticipatory eye movements ahead of the ball’s position at multiple time points throughout the ball’s flight toward them, rather than simply tracking the ball’s actual location. In addition, Diaz, Cooper, Rothkopf, and Hayhoe (2013) used a virtual racquetball task and found that participants made predictive eye movements to locations above where the ball would bounce and varied their predictive fixations based on the ball’s bounce speed and elasticity. Similar predictive looking to future ball bounce locations were also reported when participants watched recorded tennis matches (Henderson, 2017).

These studies provide strong evidence that people make predictive eye movements while viewing naturalistic activity. However, no previous studies have tested whether predictive looking varies as a function of event structure. Therefore, in a series of two studies that were designed as close replications of one another, we used a new anticipatory looking task, called the Predictive Looking at Action Task (PLAT), to investigate the time course of predictability. For this task, participants’ eyes were tracked as they watched movies of an actor performing an everyday activity that consisted of sequences of goal-directed actions. Participants were not told to engage in any explicit task other than paying attention to the movie. Prediction was measured based on the amount of time participants spent looking at the object the actor was about to touch during the three seconds before the actor actually contacted the object. This task therefore allowed predictive looking to be time locked to object contact and made it possible to analyze the time course of predictive looking. We hypothesized that looking to the to-be-contacted object would increase as time to object contact approached, providing an index of predictive looking.

After watching each of the movies once, participants segmented the movies into meaningful units of activity twice: once to identify the largest meaningful units of activity (coarse events) within each movie and once to identify the smallest meaningful units of activity (fine events) within each movie. The locations at which participants identified event boundaries were time-locked to their predictive looking behavior during the passive watching condition.

We hypothesized that if prediction error is higher near event boundaries, then our index of predictive looking would be effective; specifically, we predicted that looks to the target object in the interval before contact would be less frequent or would tend to be later (closer to object contact) when objects were contacted near event boundaries than when objects were contacted during the middles of events. This amounts to predicting a main effect on looks to the target object (an overall reduction), an interaction (looks to the target object shifting to be relatively later in time), or both.

## Materials and methods

The present data come from a larger study investigating oculomotor control in naturalistic event viewing. A previous report (Eisenberg & Zacks, 2016) characterized the effect of event boundaries on the size and frequency of eye movements, and on pupil diameter. All of the analyses reported here are new.

### Participants

Participants for Study 1 were recruited from the Washington University subject pool and were either given course credit or paid $10 for their time. Thirty-two people participated in the first study, but four were dropped from all analyses because of inability to calibrate the eye tracker (2), self-reported lazy eye (1), and self-withdrawal from the study (1). Therefore, data from 28 participants were included in the analyses reported here (50% female, age range 18–25 years, mean age 20.6 years). Participants for the second study were recruited from the Volunteer for Health participant registry, which is a subject pool maintained by the Washington University School of Medicine. Thirty-two participants finished Study 2, but seven were dropped from all analyses because of inability to calibrate the eye tracker (5), failure to follow instructions (1), and technical error leading to loss of data. Therefore, data from 25 participants were included in the analyses for the study (68% female, age range 22–50 years, mean age 34 years). The Washington University Human Research Protection Office approved both studies.

### Materials

Three movies of actors performing everyday activities were used in each study. The three movies used in the first study were an actor making copies and putting together a binder (349 s), an actor sweeping the floor (329 s), and an actor changing a car tire (342 s). The movies for the second study were an actor making breakfast (329 s), an actor preparing for a party (376 s), and an actor planting plants (354 s). All six of these movies were filmed from a fixed, head-height perspective, with no pan or zoom.

### Self-report measure

Before beginning the eye-tracking tasks, participants completed a demographics questionnaire that included age, gender, handedness, ethnicity, foreign language knowledge, occupational history, educational history, marital status, health status, and level of typical physical activity.

### Behavioral and oculometric measures

Participants in both studies first watched three movies without any explicit task other than to pay close attention to the movies. After watching all three movies, participants watched the three movies two more times. During these latter two viewings, they were asked to press a button whenever they believed that one meaningful unit of activity had ended and another had begun. On one viewing, they were asked to identify the smallest units that were natural and meaningful to them (fine grain segmentation); during the other repetition, they were instructed to identify the largest units that were natural and meaningful (coarse gain segmentation). For example, a typical participant might have identified a coarse unit that could be described as “making toast”, and fine units within that coarse unit that could be described as “opening a bag of bread”, “putting bread in the toaster”, and “turning on the toaster”. (Participants were not given any such descriptions or specific instructions as to what should constitute a fine or coarse unit, beyond those given above.) The order of fine and coarse segmentation was counterbalanced across participants. In addition, participants were not told anything about the event segmentation task until after they had finished watching all three movies passively in order to ensure that participants would not covertly button press in the passive condition. Participants’ consistency in identifying event boundaries was similar to that seen in other studies of event segmentation (see Additional file 1 for a description of this analysis and the results).

Throughout all of these tasks, gaze location from the participants’ right eye was tracked using an infrared eye tracker (EyeLink 1000; SR Research Ltd, Mississauga, ON, Canada) that sampled at 1000 Hz. The eye tracker camera was mounted on the SR Research Desktop Mount. Participants were instructed to keep their heads in an SR Research chin/forehead reset throughout all of the tasks to minimize head movement during the tasks. The camera was positioned 52 cm from the top of the forehead rest. The movies were presented on a 19 in (74 cm) monitor (1400 × 900 resolution, viewing distance of 58 cm from the forehead rest, viewing angle of 38.6°). Data were exported from Data Viewer software (SR Research Ltd, Mississauga, ON, Canada) into text files, which were then imported into R (R Core Team, 2014) for analysis.

Calibration of the eye tracker was conducted before beginning the study task. Participants were instructed to look at each of five to nine dots presented serially across the participant’s central and peripheral visual field. Following calibration, the measurements were validated by having the participants look at each of these nine dots again as they appeared on the screen. This validation of calibration was considered good when there was an average error of 0.50 degrees of visual angle or less and when the maximum error for any given dot was 1.00 degree or less. Calibration and validation were repeated until errors were under or very close to these cut-offs. For Study 1, the mean average error was 0.35 degrees (range = 0.18–0.54), and the mean of the maximum error was 0.73 (range = 0.43–1.0). For Study 2, the mean average error was 0.39 (range = 0.2–0.69), and the mean of the maximum error was 0.73 (range = 0.37–1.04).

### Data analysis

#### Time course of anticipatory looking

**Fig. 1:**
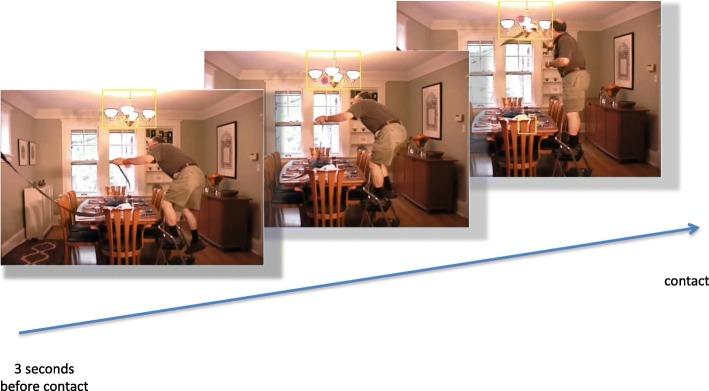
Three example frames taken from one of the movies used in Study 2. The first frame is taken from around three seconds before the actor contacted the chandelier and the third frame is taken from around the time the actor contacted the chandelier. The *yellow box* represents the interest area, which was drawn around the chandelier—the object the actor was about to contact in order to hang a streamer. The *purple dot* represents the gaze location of one participant. Here, the participant looked at the chandelier before the actor contacted it. (Participants did not see the yellow box or their own gaze location while performing the task)

Predictive looking was quantified by determining the amount of time participants spent looking at the objects the actor was about to touch over a series of time bins spanning the 3000 ms before contact. First, for each movie, an experimenter identified all of the time points at which the actor came into contact with an object. Dynamic interest areas capturing the 3000 ms before contact through 1000 ms after contact were then placed around each contacted object. Interest areas were placed using the following rules: (1) all interest areas were rectangular in shape; (2) no interest areas were allowed to overlap in time and space; (3) if potential interest areas overlapped, only the first interest area was kept; (4) if the actor contacted an object by touching it with another object, the object in direct contact with the actor was considered the object of interest (e.g., if the actor put a bowl on the counter, the bowl was considered the object of interest); (5) only objects that were fully onscreen when contacted were considered objects of interest; (6) if the longest dimension of an object was smaller than 105 pixels (visual angle of 2.9°), the interest area was created around the entire object, and if the longest dimension of an object was larger than 105 pixels, the interest area was created around the part of the object that the actor contacted (e.g., the handle of a large refrigerator); and (7) for objects smaller than 48 pixels (visual angle of 1.3°) on any side, interest areas were created with a minimum size of 48 pixels per side (see Fig. 1 for an example movie frame with an interest area highlighted). For the movies in the first study, there were 51, 45, and 29 dynamic interest areas for the binder, sweeping, and tire movies, respectively. For the movies in the second study, there were 49, 48, and 34 dynamic interest areas for the breakfast, party, and plants movie, respectively.

Once the dynamic interest areas were identified for each movie, the eye tracking data from the three seconds before contact until one second after contact were divided into six 500 ms time bins. Then, we calculated for each subject how long their gaze fell within the interest area for each of the time bins. This metric was calculated separately for each time bin and was not a cumulative measure of looking over time. This variable was the dependent measure in the reported mixed-effects model analyses. The main analyses examined the three seconds before object contact, but figures displaying the time course of looking during the second after contact are included in Additional file 1: Figures S1 and S2. To determine how looks to the target object varied as time to object contact approached, the lme4 and lmerTest packages in R (Bates, Maechler, Bolker, & Walker, 2015; Kuznetsova, Brockhoff, & Christensen, 2014) were used to compare nested mixed-effects models to determine whether including the fixed effect of time bin explained significant additional variance in the dependent variable. The *effects* package in R (Fox, 2003) was used to estimate the fixed effects for plotting and to calculate confidence intervals for the fixed effects in the linear mixed effects models.

#### Time course of predictive looking around event boundaries

**Fig. 2:**
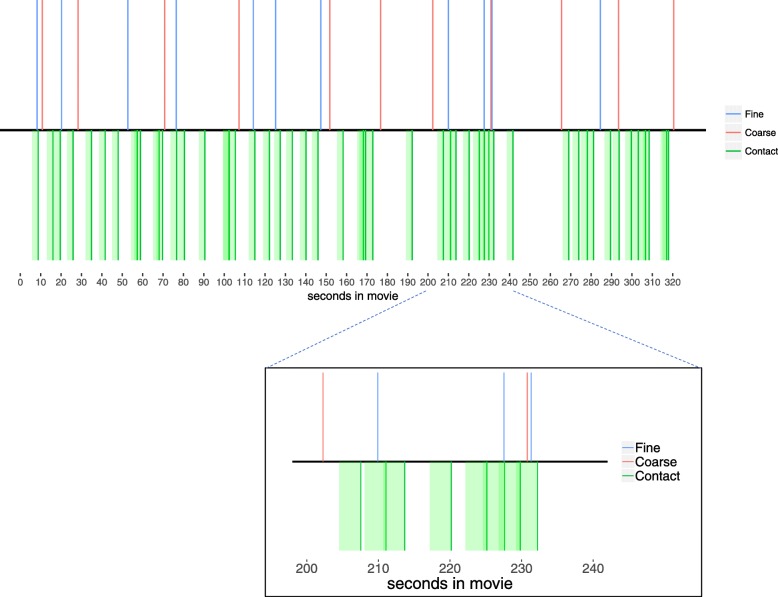
The time course of one movie for a randomly chosen participant from Study 2. The x-axis displays the time in the movie in seconds. The *blue* and *red lines* depict fine and coarse boundaries, respectively. The *dark green lines* are the times of object contact. The *light green shading* depicts the 3000-ms interest period during which looking was measured. The inset zooms in on 40 s in the same movie to better show the relationships between event boundaries and object contacts. As is evident from this time course, it was possible for there to be multiple fine and course event boundaries during the three seconds before object contact. In addition, it was possible for there to be only fine, only coarse, or both fine and coarse boundaries within the three seconds before a given object contact

To test the effect of event boundaries on predictive looking, we coded whether each object contact happened within 3000 ms of a coarse boundary or within 3000 ms of a fine boundary. This was done separately for each participant, based on the segmentation data from their subsequent viewing of the movie. (Only the initial viewing eye tracking data were analyzed to exclude contamination from the cognitive operations necessary for the segmentation task.) Because the locations of event boundaries were identified individually by each participant, object contact could be near an event boundary for some participants but not for other participants. In addition, it was possible for there to be multiple event boundaries within the time window around object contact. An example time course from a randomly chosen participant is displayed in Fig. 2.

Mixed-effects models were analyzed to determine whether being near a fine or coarse event boundary reduced the overall amount of looking to the target object (a main effect of event boundary condition) or shifted when looks to the target object occurred (an interaction between event boundary condition and time relative to contact). (If the temporal window before object contact were sufficiently large, one would expect no effect for the earliest timepoints, before predictive looking is possible, with the effect building as time to contact approached.) Again, the *effects* package in R (Fox, 2003) was used to estimate fixed effects and confidence intervals. For Study 1, 4.1% of the interest areas were classified as being near a coarse event boundary, 41.1% were classified as being near a fine event boundary, and 15.2% were classified as being near both a coarse and a fine boundary. For Study 2, 5.5% were classified as being near a coarse event boundary, 34.8% were classified as being near a fine event boundary, and 17.6% were classified as being near both a coarse and a fine boundary. Follow-up analyses were also conducted to control for the size of the interest area drawn around each contacted object in order to ensure that interest area size did not drive the event boundary effects.

## Results

### Time course of predictability

**Fig. 3:**
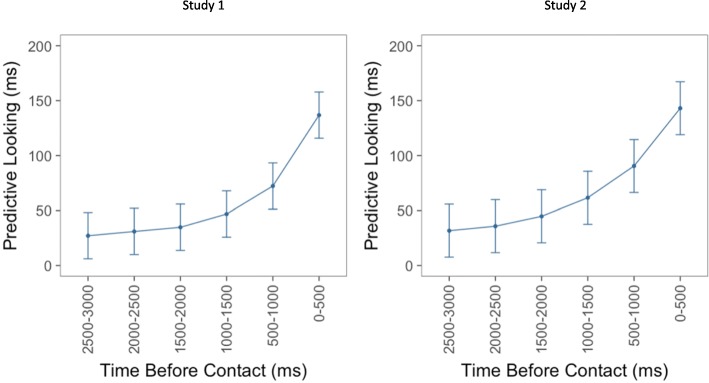
The panel on the *left* displays the results of the linear mixed effects model for the first study. The y-axis displays the beta values representing the amount of time participants spent looking at the target object during each of the six 500-ms bins. The panel on the *right* displays the same information for the second study. The x-axis for both figures displays the six bins, where the leftmost bin on each x-axis represents the time farthest away from contact and the rightmost bin represents the time closest to contact. *Error bars* depict 95% confidence intervals

Looking to the target object increased over the three seconds before contact. Specifically, for both studies, there was a main effect of time bin (Study 1, χ^2^ = 2183.1, df = 5, *p* < 0.001; study 2, χ^2^ = 1858, df = 5, *p* < 0.001), suggesting that looks to target objects increased as object contact approached. Figure 3 displays the amount of time participants spent looking at target objects within each of the six 500-ms bins during the three seconds before the actor contacted the target object.

### Time course of predictability around event boundaries

**Fig. 4:**
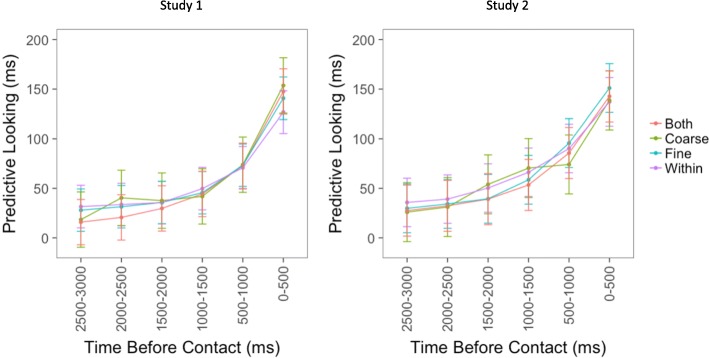
The panel on the *left* displays the results of the linear mixed effects model for the first study. The y-axis displays the beta values representing the amount of time participants spent looking at the target object during each of the six 500-ms bins. The panel on the *right* displays the same information for the second study. The x-axis for both figures displays the six bins, where the leftmost bin on each x-axis represents the time farthest away from contact and the rightmost bin represents the time closest to contact. Each *colored line* represents the amount of time participants spent looking at the target object for each boundary type. *Error bars* depict 95% confidence intervals calculated based on between-subjects effects

**Fig. 5:**
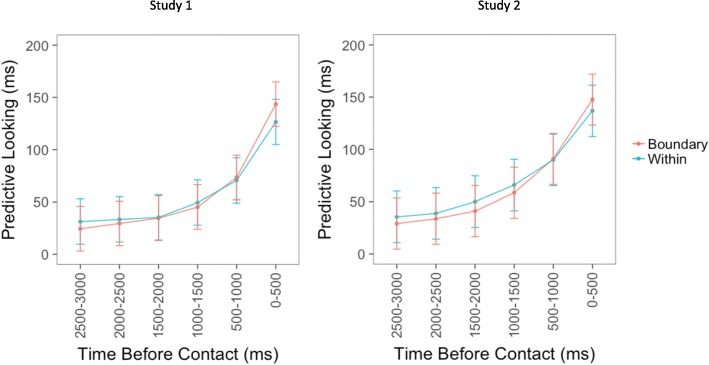
The panel on the *left* displays the results of the linear mixed effects model for the first study. The y-axis displays the beta values representing the amount of time participants spent looking at the target object during each of the six 500-ms bins. The panel on the *right* displays the same information for the second study. The x-axis for both figures displays the six bins, where the leftmost bin on each x-axis represents the time farthest away from contact and the rightmost bin represents the time closest to contact. The *red line* displays predictive looking collapsed across boundary types (fine, coarse, both fine and coarse). The *blue line* displays predictive looking when there were no event boundaries nearby (within events). *Error bars* depict 95% confidence intervals calculated based on between-subjects effects

To investigate the time course of predictability around event boundaries, mixed-effects models were tested with time bin and boundary type (within events, fine boundary, coarse boundary, both fine, and coarse boundaries) as fixed effects and item, movie, and subject as random effects. For both studies, a model with an interaction between time bin and boundary type fit the data significantly better than a model with only the main effects (Study 1, AIC = 259,606 vs AIC = 259,602, χ^2^ = 33.9, df = 15, *p* = 0.004; Study 2, AIC = 245,626 vs AIC = 245,630, χ^2^ = 25.4, df = 15, *p* = 0.04). For both studies, there was a significant main effect of bin (Study 1, F = 233.72, df = 5, *p* < 0.001; Study 2, F = 211.75, df = 5, *p* < 0.001), and a significant interaction between time bin and boundary type (Study 1, F = 2.26, df = 15, *p* = 0.004; Study 2, F = 1.69, df = 15, *p* = 0.04). The form of the interaction is illustrated in Figs. 4 and 5: for objects contacted in the middles of events participants looked to the object relatively early, whereas for objects contacted near event boundaries they tended to look more just before object contact. The main effect of boundary type was not significant (Study 1, F = 0.95, df = 3, *p* = 0.42; Study 2, F = 1.38, df = 3, *p* = 0.25).

To determine whether boundary types differed significantly from one another, three nested models were tested: a null model containing a binary variable coding whether there was an event boundary present or not, a model with this binary variable and a variable coding for the effect of fine boundaries, and a model adding a variable coding for the effect of coarse boundaries. All three models also included interaction terms coding for the interaction of time point and boundary type. None of these models were significantly different from one another (Study 1, largest χ^2^ = 12.86, df = 12, *p* = 0.38; Study 2, largest χ^2^ = 13.41, df = 12, *p* = 0.34).^1^ Therefore, all three boundary conditions (fine, coarse, and both fine and coarse) were collapsed into a single boundary variable, as depicted in Fig. 4. For both studies, a model with an interaction between time bin and boundary type fit the data better than a model with only the main effects (Study 1, AIC = 259,605 vs 259,591, χ^2^ = 23.7, df = 5, *p* < 0.001; Study 2, AIC = 245,624 vs 245,620, χ^2^ = 14.6, df = 5, *p* = 0.01). There was a significant main effect of time bin (Study 1, F = 422.05, df = 5, *p* < 0.001; Study 2, F = 370.82, df = 5, *p* < 0.001), and again there was a significant interaction between time bin and boundary type for both studies (Study 1, F = 4.75, df = 5, *p* < 0.001; Study 2, F = 2.92, df = 5, *p* = 0.01). The main effect of boundary was again not significant (Study 1, F = 0.13, df = 1, *p* = 0.72; Study 2, F = 1.50, df = 1, *p* = 0.22).

To assess which, if any, individual time points had significant differences between the boundary and within-event conditions, we fitted mixed-effects models testing the difference for each time point. None of these were significant (Study 1, largest F = 3.03, *p* = 0.08; Study 2, largest F = 1.04, *p* = 0.31).

Follow-up analyses found that the size of interest areas differed significantly between boundary and within-event conditions for Study 1, but not for Study 2 (Study 1, Within = 5470.9 pixels, sd = 2631.5, Boundary = 5731.5 pixels, sd = 2552.3, *t* = − 7.1, *p* < 0.001; Study 2, Within = 3877.5 pixels, sd =2882.8, Boundary = 3864.7 pixels, sd = 2632.6, *t* = 0.31, *p* = 0.75). Therefore, to ensure that interest area size did not drive the event boundary effects, mixed-effects models that controlled for the size of each interest area were tested. The same pattern of results as reported above was found for both studies. There were main effects of interest area size (Study 1, F = 17.56, df = 1, *p* < 0.001; Study 2, F = 27.17, df = 1, *p* < 0.001) and of time point (F = 422.05, df = 5, *p* < 0.001; Study 2, F = 370.82, df = 5, *p* < 0.001), and there was a significant interaction of time point and boundary type (Study 1, F = 4.75, df = 5, *p* < 0.001; Study 2, F = 2.92, df = 5, *p* = 0.01). The main effect of boundary was not significant for either study (Study 1, F = 0.10, df = 1, *p* = 0.76; Study 2, F = 1.55, df = 1, *p* = 0.21).

In sum, both experiments showed an interaction between time and boundary condition, such that around event boundaries compared to within events, participants looked less at the target objects early and looked more during the 500 ms before object contact. There was no overall reduction in looking to the target object. In other words, event boundaries were associated with a shift in looking such that looks to the target location occurred closer to the point at which the object would be contacted. There was no evidence that coarse and fine boundaries differed from each other, and in neither experiment could the effect be statistically localized to any individual time points. In addition, these effects held even after controlling for the size of the interest areas.

## Discussion

The current studies introduce the PLAT as a tool for investigating the time course of predictive looking and provide the first demonstration of the dynamics of predictive looking during viewing of a naturalistic sequence of activities. In both studies, the amount of time participants spent looking at a target object increased as the actor came closer to contacting the object. This result provides validation that this looking behavior can be used as a noninvasive measure of prediction during ongoing comprehension.

After determining that the PLAT could be used to investigate predictive processing, it was used to study the dynamics of predictability around event boundaries. In both studies, the amount of time participants spent looking at the object the actor was about to contact increased progressively during the three seconds before contact. In addition, there was a significant interaction such that looks to the target object happened later around event boundaries than within events; in other words, eye movements were less anticipatory of the object contact to come. All of the results replicated across the two studies, providing strong evidence for these effects.

Our interpretation of this interaction is that in the middle of an event it is easier to predict what will happen two to three seconds ahead, so viewers’ eyes are somewhat likely to jump ahead to the to-be-contacted object. For example, in Fig. 1, once the actor steps on the ladder and reaches up toward the chandelier with the streamer in his hand, it is fairly clear which object the actor is about to touch because there are no other objects nearby. When this happens, their eye may have moved on by the time the actor’s hand actually reaches the object, leading to decreased looking times right before contact. In contrast, near an event boundary it is more difficult to predict two to three seconds ahead, so the eye is more likely to reach the object being manipulated just before the actor’s hand arrives, leading to increased looking times right before contact. These effects are not huge but they appear robust.

The two studies described here were purposely designed as close replications of one another in order to determine the reproducibility of the results. However, while the design of the studies was nearly identical, the populations and movies used in the studies differed. In the first study, participants were undergraduates recruited from the university’s participant pool, whereas in the second study, the participants were recruited from the general population of St. Louis and included a much more diverse age range. In addition, completely different movies were used in the two studies to ensure that the results were not specific to particular sequences of naturalistic activity, but instead were generalizable to other sequences of actions. The pattern of results from the two studies was almost identical, providing strong evidence for the findings reported here.

These results, and this interpretation, are consistent with previous studies investigating prediction around event boundaries using explicit measures. In three studies, Zacks et al. (2011) asked participants to watch similar movies of everyday activities to those used in the present studies. Each movie was paused eight times, four times around event boundaries and four times within events and participants either made forced choice two-alternative decisions about what would happen five seconds later in the movie or they made yes–no decisions about whether one image would appear five seconds later in the movie. The authors found that participants were more accurate in making predictions when the movies were paused within events than when they were paused right before event boundaries.

We initially predicted that the presence of an event boundary might be associated with lower predictive looking overall, a main effect, in addition to the interaction observed. One possibility is that this main effect would have been observed if we had looked farther back in time before each object. As can be seen in Figs. 3 and 4, the largest difference between the conditions appears to be at the earliest timepoints. In the explicit prediction study of Zacks et al. (2011) a difference was found for predictions of five seconds in the future. However, a forced choice task is very different to making open-ended predictions by looking around a visual space. Therefore, it is possible that prediction would be equally bad around event boundaries and within events as object contact becomes farther away in time, especially since participants in the current study spent an average of less than 50 milliseconds looking at the target object from 2500 to 3000 milliseconds before contact.

It is also possible that the lack of a main effect of boundary type is due in part to the large difference in the number of observations for each boundary condition. As noted above, only 4.1 and 5.5% of the observations occurred near coarse boundaries, which likely explains the large confidence intervals for this condition. However, this difference in number of observations cannot fully explain the results. First, the results presented above were obtained using linear mixed effects modeling, which took into account the different numbers of observations among the conditions. In addition, when the boundary conditions were collapsed into within events versus around event boundaries, the number of observations in each condition were more similar (60.4 and 57.9% of observations were around event boundaries for the two studies). Therefore, it is unlikely that additional observations for the coarse boundary condition would have dramatically altered the results.

The size of interest areas differed significantly between the within-event and boundary conditions for Study 1, and although the results of the models held even when controlling for the effect of interest area size, it is necessary to consider reasons for the differences between boundary conditions. One possibility is that when larger objects are contacted and moved around on screen, more movement and other visual changes occur compared to when smaller objects are contacted. Previous studies have found that low-level visual changes in movies can predict the locations of event boundaries (Hard, Tversky, & Lang, 2006; Speer, Swallow, & Zacks, 2003.; Zacks, 2004; Zacks et al., [Bibr CR24][Bibr CR26]; Zacks, Kumar, Abrams, & Mehta, 2009a), providing some evidence that people might be more likely to identify event boundaries when larger objects are contacted. However, follow-up analyses found that, across participants, most object contacts were not consistently identified as either within-events or around boundaries. For Study 1, across object contacts, an average of 11.1 (sd = 6.8, range = 0–28) participants identified object contacts as within-events and 16.9 (sd = 6.8, range = 0–28) participants identified object contacts as boundaries. In addition, for Study 1, the correlation between the size of an interest area and the number of participants who identified an event boundary near that interest area was only 0.1 (*p* = 0.27). Therefore, the size of an interest area did not significantly predict whether participants would identify an event boundary nearby.

Furthermore, because some objects were contacted multiple times during each movie (Study 1, 16 objects; Study 2, 24 objects), it was possible to determine whether features of an object strongly determined where people identified event boundaries by determining whether participants consistently identified event boundaries near these repeatedly contacted objects. The results suggest that this was not the case, as there was little consistency across repeated contacts of the same object. For Study 1, repeatedly contacted objects were consistently classified as near event boundaries 41.7% of the time (sd = 0.20, range = 12.5–87.5%), were consistently classified as within-event 12.1% of the time (sd = 0.12, range = 0–37.5%), and were classified inconsistently 46.2% of the time (sd = 0.15, range = 12.5–75%). For Study 2, repeatedly contacted objects were consistently classified as near event boundaries 33.5% of the time (sd = 0.27, range = 0–87.5%), consistently classified as within-event 23.67% of the time (sd = 0.19, range = 0–62.5%), and inconsistently classified 42.83% of the time (sd = 0.14, range = 12.5–70.8%). Therefore, neither the size of the interest areas nor other features of the objects themselves fully drove the boundary condition results. However, future studies that use interest areas that are all the same size are necessary to achieve complete confidence that the event boundary effects reported here are not due, in any way, to differences in the size of the interest areas.

In addition to its utility in the current studies, the PLAT has strong potential for utilization in other studies investigating predictive processing. The PLAT allows for the collection of large amounts of data in a short amount of time, as each of the five- to six-minute movies contained between 29 and 51 target objects and 3000 data points were analyzed for each of these trials. Although this study was not designed to investigate individual differences in predictive looking, the large amount of data that can be collected using the PLAT positions it as a potentially powerful individual differences measure. If individuals vary in their time course of predictive looking, performance on the PLAT might correlate with other cognitive measures such as working memory or executive function. It would be informative to determine whether predictive looking behavior is fully explained by other cognitive abilities or whether it is a separate cognitive ability in a similar way as working memory is at least partially independent of executive processing.

The PLAT also has potential for studying predictive processing in populations that are unable to perform explicit prediction tasks. For example, the task can be used with infants or very young children, who would not be capable of performing an overt prediction task. The task could also be used to investigate predictive processing in clinical populations who may not have the verbal or motor ability to complete a prediction task that requires overt responses.

## Conclusions

People engage in prediction in almost every moment of the day, and understanding how predictability varies over time is integral to understanding how people comprehend ongoing activity. Using a novel predictive looking task, the two current studies extended previous research on the time course of predictability, finding that participants engaged in less predictive looking around event boundaries than within events at time points furthest from contact and participants engaged in more predictive looking around event boundaries immediately before contact. These results are consistent with previous studies that found decreases in predictability at times of greatest change in the environment. The two current studies also demonstrated the utility of the PLAT as a sensitive measure of the time course of prediction, and the PLAT can easily be extended to naturalistically study prediction ability in healthy adults, clinical populations, infants and young children, and even non-human primates.

## Additional file

Additional file 1: Supplemental material. (DOCX 588 kb)

## Abbreviations

EST: Event Segmentation Theory
PLAT: Predictive Looking at Action Task

## Footnotes

1 To determine whether looking behavior differed depending on whether there was an event boundary before versus after object contact, a new model that differentiated between event boundaries that occurred before or after object contact was tested against the simpler model described above. The new model did not provide better explanatory power than the original, simpler model (Study 1, AIC for original model = 226,872, BIC for original model = 227,090, AIC for new model = 226,878, BIC for new model = 227,238; Study 2, AIC for original model = 214,268, BIC for original model = 214,338, AIC for new model = 214,267, BIC for new model = 214,360), suggesting that it was not necessary to differentiate between event boundaries that occurred before versus after object contact.
