# Supplementary material for: Dynamic prediction during perception of everyday events
Source: Cogn Res Princ Implic. 2018 Dec 29;3:53. doi: 10.1186/s41235-018-0146-z (PMC6311167; doi:10.1186/s41235-018-0146-z)
Supplement: Supplementary file 1 — Supplemental material. (DOCX 588 kb) [file 41235_2018_146_MOESM1_ESM.docx]

**Supplementary Materials**

Event Segmentation Consistency Analysis:

To determine how consistent participants were in their specifications of fine and coarse event boundaries, a segmentation agreement score was calculated for each participant. Segmentation agreement is a measure of the similarity between each participant’s segmentation and the segmentation of the group as a whole. We calculated segmentation agreement using the method described by Kurby and Zacks (2011). First, time in each of the movies was divided into one-second bins. Then, group norms for fine and coarse boundaries were calculated by determining the proportion of participants who identified an event boundary within each bin. Each participant’s fine and coarse segmentation data was correlated with their respective group norm, and the resulting correlations were scaled to 0-1, based on the maximum and minimum correlation possible given how many boundaries were identified by each participant. Segmentation agreement scores for fine and coarse boundaries were then averaged to obtain an event segmentation consistency for each boundary type. For Study 1, segmentation agreement was 0.56 for coarse boundaries and 0.67 for fine boundaries. For Study 2, segmentation agreement was 0.54 for coarse boundaries and 0.59 for fine boundaries. These average agreement scores are consistent with those reported in other studies.

Figure S1: The panel on the left displays the results of the linear mixed effects model for the first study, including the 1000 ms after contact. The panel on the right displays the same information for Study 2. The x-axis on both figures displays the eight bins, with the first six bins representing the 3000 ms before contact and the last two bins representing the 1000 ms after contact. The y-axis on both figures displays the amount of time participants spent looking at the target object. Error bars depict 95% confidence intervals.

Figure S2: The panel on the left displays the results of the linear mixed effects model for the first study when boundary type is included in the model. The panel on the right displays the same information for Study 2. The x-axis on both figures displays the eight bins, with the first six bins representing the 3000 ms before contact and the last two bins representing the 1000 ms after contact. The y-axis on both figures displays the amount of time participants spent looking at the target object. Error bars depict 95% confidence intervals.

**References**

Kurby, C. A., & Zacks, J. M. (2011). Age differences in the perception of hierarchical structure in events. *Memory & Cognition*, *39*(1), 75–91. http://doi.org/10.3758/s13421-010-0027-2
